# Frequency and associated factors of axillary web syndrome in women who had undergone breast cancer surgery: a transversal and retrospective study

**DOI:** 10.1186/s40064-015-0889-7

**Published:** 2015-03-05

**Authors:** Kassandra Ferreira Pessoa Fukushima, Luana Aroucha Carmo, Adriana Carvalho Borinelli, Caroline Wanderley Souto Ferreira

**Affiliations:** Departamento de Patologia, Centro de Ciências da Saúde, Universidade Federal de Pernambuco, Avenida Professor Moraes Rego, 1235, Cidade Universitária, Recife, PE CEP: 50670-901 Brazil

**Keywords:** Vascular system injuries, Axilla, Postoperative complications, Lymph node excision

## Abstract

**Background:**

Breast cancer is the most common malignancy among women. Surgical treatment is an essential part of therapy, which still includes chemotherapy, radiotherapy and hormone therapy. The increase in early cancer detection and less aggressive treatment has made longer survival rates possible for women with this neoplasia. Morbidities after treatment have subsequently aroused particular interest in the scientific community in order to minimize their effects and provide increased quality-of-life for these patients. The present study aimed at investigating one of these morbidities: axillary web syndrome, which occurs after axillary surgical management.

**Methods:**

From December 2011 to September 2012, according to the inclusion and exclusion criteria, 97 patients, who had been surgically treated for breast cancer, were enrolled, interviewed, and submitted to a specific physical exam. An investigation of the axillary cords, characteristic of this syndrome, was performed in all patients.

**Results:**

The axillary web syndrome was diagnosed in 28.86% of the women. Higher risk of triggering the syndrome has been associated with younger age (21.7%), longer time between first treatment and data collection (29.3%), greater number of resected lymph nodes (149.7%) and surgical management medical teams (113.2%).

**Conclusions:**

One can conclude that axillary web syndrome was associated with younger age, greater time elapsed since surgery, surgical management of medical staff and number of resected lymph nodes.

Further studies are needed to review prior-to-surgery and post-operative follow-up, to properly assess the effects of surgery in the axilla on homeostatic balance, not only in the ipsilateral upper limb, but also assess their compensatory consequences throughout the body.

## Introduction

Breast cancer is considered a global public health problem due to its increasing incidence and associated socioeconomic implications (Brasil. Instituto Nacional do Câncer Jose Alencar Gomes da Silva. Coordenação Geral de Ações Estratégicas 2011). It is the most frequent cancer among women in the world (Siegel et al. 2012; Canadian Cancer 2012; Freitas-Júnior et al. 2012).

As an immediate consequence of the increased diagnosis of this neoplasia, a great therapeutic arsenal has been used, which includes surgical, radiotherapeutic and clinical treatments (chemotherapy, hormonal therapy, among others) (Ewertz & Jensen 2011). As a result of such treatments, a variety of clinical problems have followed that may have functional impacts (Hellman & Harris 2002).

Surgical trauma and/or radiotherapy can result in deficiencies in the ipsilateral upper limb, functional limitations and other morbidities including pain, stiffness, lymphedema, seroma, axillary web syndrome (AWS), decreased strength and range of motion (ROM), decreased tolerance to everyday activities (Isaksson & Feuk 2000; Springer et al. 2010), and grasping weakness of the hand, postural changes and increased sensitivity of the thoracic wall, cervical or anatomical region corresponding to the upper trapezius muscle (Hellman & Harris 2002).

The morbidity called axillary web syndrome, presents itself as a taut, stretched band underneath the skin, sometimes called a cord (Koehler 2009) or cord of lymphedema. It originates in the axilla and extends to the medial and upper portion of the arm to the anterior portion of the elbow (Moskovitz et al. 2001).

It is assumed that these fibrous bands could be sclerified lymphatic vessels (Hellman & Harris 2002), since it is a condition that can occur after stopping the lymphatic flow in the armpit, induced by the dissection of axillary lymph nodes, by the dissection of the sentinel lymph node (the first lymph node to receive lymphatic drainage of a tumor), trauma, or even by the cancer itself (Koehler 2009; Biazús 2000).

Cord biopsy of a small number of patients has indicated dilated lymphatic vessels, fibrosis of the lymphatic vessels and venous thrombosis (Koehler 2009; Moskovitz et al. 2001), although this latter find is rare in women with breast cancer (De Martino et al. 2012; Lovely et al. 2012). The observation of fibrin clots in superficial veins and axillary lymphatic vessels, submitted to biopsies, suggests that the trauma to lymphatic vessels and veins, stasis and hypercoagulability are implicated in the genesis of this syndrome. Thus, pathological and anatomical evidence help support the hypothesis of the angiolymphatic origin of AWS (Moskovitz et al. 2001).

Little is known about the factors that could predispose someone to this syndrome. The injury of axillary lymph nodes seems, according to the foregoing, to occupy the central place in its genesis. This observation has been corroborated by a Brazilian study, which found an association between the presence of AWS with a greater number of removed and compromised lymph nodes (Bergmann et al. 2012). Another aspect to be noted is the patient’s age at diagnosis and treatment of breast cancer; research has shown that, in young women, the intensity of symptoms in the immediate postoperative period tends to be exacerbated (Steegers et al. 2008; Tasmuth et al. 1995). In addition, the time elapsed after the surgical event seems to influence the detection of AWS, as the syndrome can occur not only in the immediate postoperative period, according to some authors (Lacomba et al. 2009).

The exact frequency, origin, clinical presentation, evolution and treatment of axillary web syndrome are still undefined (Leduc et al. 2009) and, thus, there is a lack of formal guides on which therapeutic interventions can be based (Fourie & Robb 2009).

The present research aims to determine the frequency and the factors associated with axillary web syndrome in women who have undergone surgical treatment of breast cancer, which involved the axilla in the Cancer Hospital of Pernambuco.

## Methods

The study was conducted at Cancer Hospital of Pernambuco (CHP), in the Department of Mastology and in the Service of Medical Records and Statistics (SMRS). Joining the study was voluntary and all patients who wished to participate signed an informed and free consent form. The research was approved by the Research Ethics Committee of the hospital under registration CAAE 03260172447–11 on 09 August 2011.

The first phase of the study was transversal, observational, analytical, with comparison of groups, concerning the detection of axillary web syndrome in order to meet the objective of this study. This was followed by a retrospective, descriptive study and was restricted to collecting the data of histopathological and therapeutic features of the breast cancer that were recorded in the medical record, for investigation of a possible causal relationship between axillary web syndrome and tumor characteristics or the therapy applied.

The population was composed of female patients, older than 18 years, served in the Department of Mastology of the Cancer Hospital of Pernambuco between December 2011 and July 2012 and which had been submitted to surgical treatment for breast cancer with which involved the axilla.

To determine the sample size, an incidence of axillary web syndrome equal to 28.1% was applied, which refers to the study by Bergmann et al. (2012), the only recent Brazilian study located with determination of this parameter, and 6%, which refers to a study by (Moskovitz et al. 2001), being the smallest incidence found and that which was first reported. This percentage was applied to the formula of (Whitley & Ball 2002) from which the sample size was obtained, varying between approximately 94 patients (for a significance level of 0.05 and test power equal to 90%) and 162 patients (for a 0.01 significance level and test power equal to 95%). Data were collected from 131 patients, which corresponded to a test power equal to 99.3% (at a significance level of 0.05). Upon applying the inclusion and exclusion criteria, the resulting sample included 97 patients.

Independent variables studied were the following: age, body mass index (BMI), clinical stage, number of lymph nodes removed, number of compromised lymph nodes, mammary lateralization, time interval from diagnosis to surgical treatment, time interval between surgery and the interview, the time interval between the first treatment and the interview, types of surgery (modified radical mastectomy, simple mastectomy, sectionectomy, quadrantectomy associated or not with axillary surgery – conventional dissection or sentinel lymph node examination), chemotherapy and radiotherapy.

The dependent variable was axillary web syndrome, characterized as presence of strands originating in the axilla of women submitted to surgical treatment of breast cancer that involved the axilla. In this study only the presence of strands was considered, regardless of the presence of pain upon palpation or limitation to the ipsilateral upper limb movement.

The patients included were submitted to questionnaires to record the symptoms related to breast cancer and its treatment.

Weight and height were measured for each patient. An examination for similar structures to the strands that originate in the axilla and that appear with the movement of abduction, extension and external rotation of the shoulder joint, for the diagnosis of AWS, was done in all women included.

From consulting the medical chart in the files of the Cancer Hospital of Pernambuco, histopathological data for the breast cancer and information on therapy applied were obtained.

The data were organized using the spreadsheet program Excel®, and analyzed with the Statistical Package for Social Sciences (SPSS®), version 20.0. The variables in nominal or ordinal scales were presented in the form of tables, containing absolute and relative frequency distribution. The quantitative variables were expressed as mean and standard deviation from the average.

In the comparison between groups, the Student's t test was applied to the difference of averages. In addition, chi-squared and Fisher exact tests were used for comparison of proportions. A significance level of 0.05 was adopted in all inferential tests.

Subsequently, a Spearman multivariate analysis of factors was applied in order to determine the factors that exerted the greatest influence on the determination of AWS. With the values of the variables admitted as factors, normal and rotated variance matrices were built. The following values were calculated: a) the Kayser-Meyer-Olkin coefficient, for comparison between the variation coefficients of each factor with AWS and the general coefficient of the set of factors for determination of AWS; b) Bartlet sphericity test, to verify the significance of all the correlations made to the calculation matrix and c) load analysis of factors (communalities).

For the factors with significance in multivariate analysis, the odds-ratio was calculated, whose confidence interval was 95%, assuming the 0.05 significance level for rejection of the null hypothesis of association among variables and the occurrence of AWS.

## Results

Axillary web syndrome was found in 28 [28.86%] women. When classified according to age, it was found that patients with AWS had an average age of 50.54 ± 2.10 years [95% CI = 46.22-54.84 years]. For the group without AWS [69; 71.14%], this value equaled the 57.58 ± 1.72 years [95% CI = 54.16-61.00 years], and this difference was significant [Table 1].

**Table 1:** **Distribution of mean, standard error of mean, confidence interval of age, according to groups – Cancer Hospital of Pernambuco – December 2011-July 2012**

**Age**	**AWS (cords)**		**Total**
	**Present (n=28)**	**Absent (n=69)**	
Mean ± standard error of the mean*	50.54±2.10	57.58±1.72	55.55±1.40
95% CI	46.23 - 54.84	54.16 – 61.00	52.78 = 58.32
Median	51.00	57.00	53.00
Minimum	29	29	29
Maximum	67	92	92

Note: *- Student t test for differences in means; p<0.001.

In relation to the type of surgery that had been applied, there were 69 [71.13%] cases of modified radical mastectomy, in similar percentages of women with AWS [71.43%] and without AWS [71.01%] [Table 2].

**Table 2:** **Distribution of information related to the tumor and treatment of breast cancer of 97 women according to the presence and absence of AWS (cords)-Cancer Hospital of Pernambuco – December 2011-July 2012**

**Variables related to the tumor and treatment**	**AWS (cords)**				**Total (n=97)**		**P Value**
	**Present (n=28)**		**Absent (n=69)**				
	**n**	**%**	**n**	**%**	**n**	**%**	
Mamary lateralization							0.701^†^
Right	13	46.43	35	50.72	48	49.49	
Left	15	53.57	34	49.28	49	50.51	
Clinical stage of the breast cancer at the time of diagnosis							0.910^†^
Initial	19	67.86	46	66.67	65	67.01	
Advanced	9	32.14	23	33.33	32	32.99	
Type of surgery^‡^							0.856^†^
Modificed radical mastectomy	20	71.43	49	71.01	69	71.13	
Quadrantectomy with axillary dissection	5	17.86	9	13.04	14	14.43	
Mastectomy with examination of SLN	3	10.71	6	8.70	9	9.28	
Quadrantectomy with examination of SLN	-	-	5	7.25	5	5.16	
Non-surgerical therapy^¶^							0.791^†^
Not started	15	53.57	39	56.52	54	55.67	
Neoadjuvant chemotherapy	8	28.57	13	18.84	21	21.65	
Adjuvant chemotherapy	5	17.86	17	24.64	21	21.68	
Adjuvant radiotherapy	3	11.1	9	13.0	12	12.5	

Legend: * - For the variables were considered, exclusively, the examination of the sentinal lymph node and axillary dissection.

P Value calculated by: * Fisher exact test; ^†^ - chi-squared test.

^‡^ - Chi-squared test comparing modified mastectomy to quandrantectomy with axillary dissection.

^¶^ - Percentages calculated based on the total number of patients of each group, as the same patient may undergo more than one treatment.

A higher frequency of AWS was identified in therapies in which axillary dissection was performed [25; 89.28%], as well as in cases where non-surgical therapy had not yet been initiated. Such differences, however, were not significant [Table 2].

**Figure 1:**
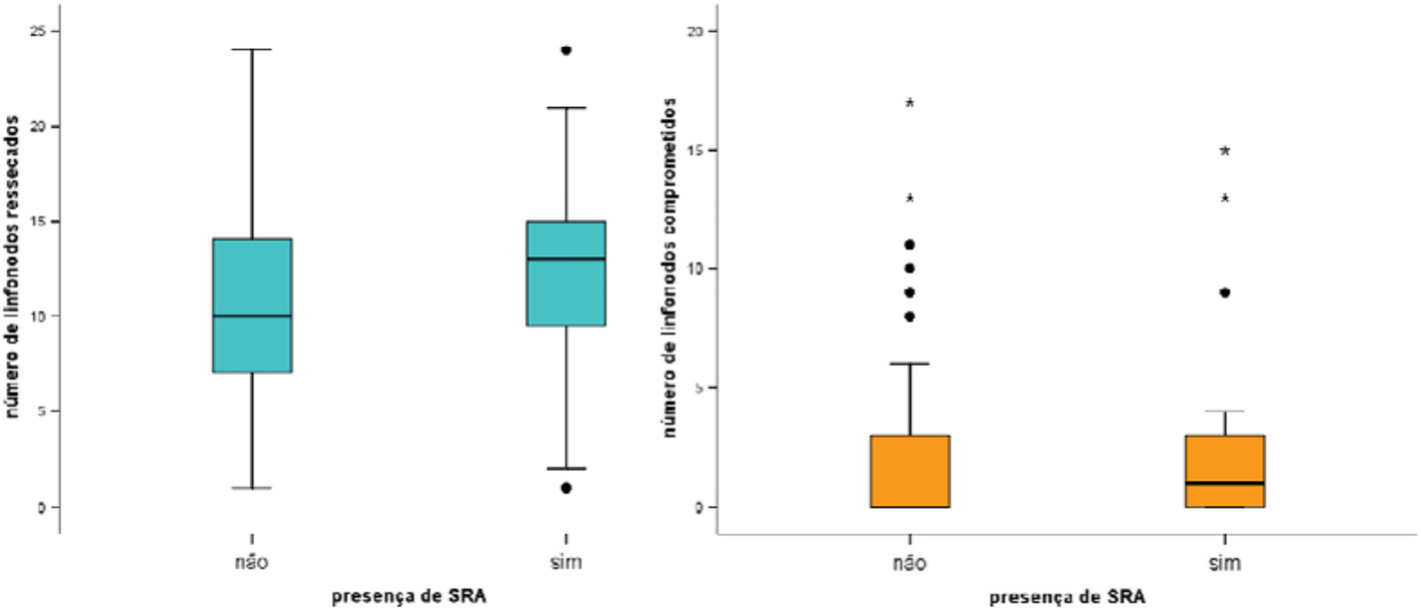
**Box-plots of the number of lymph nodes removed and compromised, according to presence of AWS-Cancer Hospital of Pernambuco-December 2011-July 2012.** Note: Student t test p=0.779.

Among the 97 women, the average number of lymph nodes removed was 12 ± 1, independent of the presence of AWS, ranging from one to 24 lymph nodes, with a median of 11 lymph nodes. When considering the group with AWS, the average number of lymph nodes removed equaled 12 ± 1, of which on average 2 ± 1 were compromised, while in the group of women without AWS, the average number of lymph nodes removed equalled to 12 ± 2, of which 4 ± 2 were compromised. These differences did not reach statistical significance [Figure 1].

In Table 3, distributions were found in the time periods that passed between diagnosis and surgical and non-surgical treatment, in relation to the admission of patients to the research. Although all the average times investigated were longer for the group of women without AWS, there were no significant differences in any parameter. The interval of elapsed time between the evaluated parameters most often reached a maximum of 60 days, regardless of the presence of AWS, there is no significant difference between the groups.

**Table 3:** **Distribution of means and the frequency of elapsed time intervals between procedures that were applied to 97 women according to presence and absence of AWS (cords)-Cancer Hospital of Pernambuco – December 2011-July 2012**

**Temporal distribution between procedures**	**AWS (cords)**				**P Value**
	**Present (n=28)**		**Absent (n=69)**		
	**Mean ±SEM**	**95% CI**	**Mean ±SEM**	**95% CI**	
From diagnosis to surgerical treatment (days)	75.6±14.4	45.91-105.2	95.0±23.7	47.6-142.5	
Up to 60 days n (%)	19 (67.8)		42 (60.9)		0.603
More than 60 days n (%)	9 (32.2)		27 (39.1)		0.342
From surgery to interview (days)	263.0±151.8	0.0-574.5	362.8±124.8	113.8-611.8	
Up to 60 days n (%)	24 (85.7)		54 (78.3)		0.649
More than 60 days n (%)	4 (14.3)		15 (21.7)		0.296
From the first treatment to interview (days)	329.8±158.5	4.0-655.7	406.3±123.6	159.6-652.9	
Up to 60 days n (%)	17 (60.7)		42 (60.9)		0.731
More than 60 days n (%)	11 (39.3)		27 (39.1)		0.582

Legend: SEM – standard error of the mean p Value calculated from the Student t test for differences in means.

**Figure 2:**
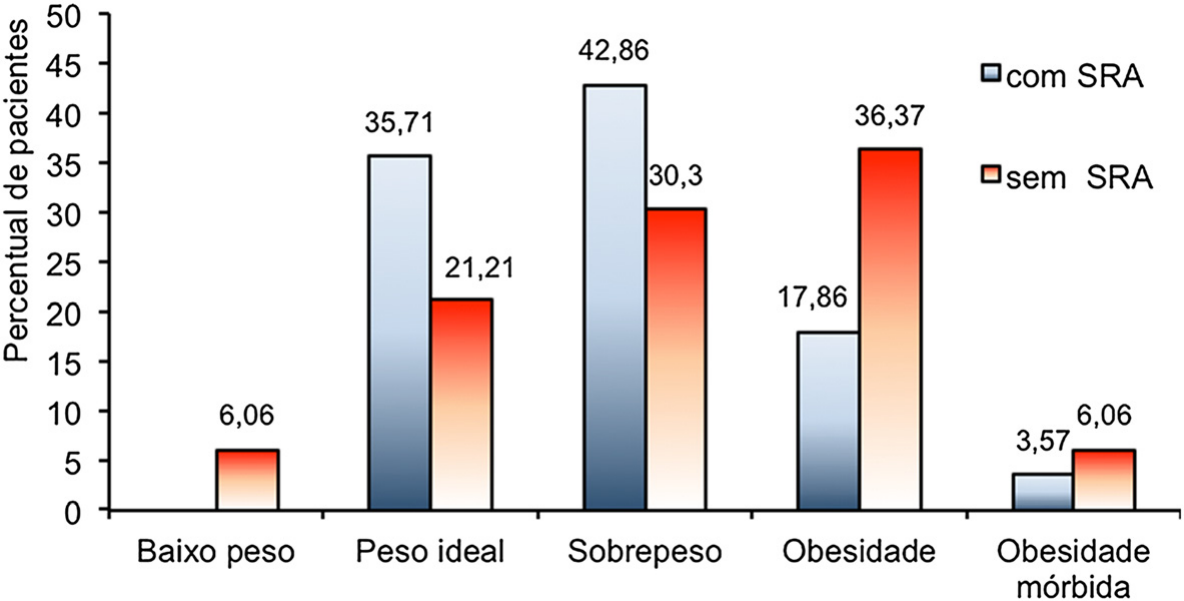
**Distribution of the classification by BMI according to diagnosis of AWS -Cancer Hospital of Pernambuco – December 2011-July 2012.** Note: p value calculated by the Snedecor F test; p=0.605; *Two (2.061%) patients did not have their body weight measured.

As for the body mass index [BMI], it was found that obesity was more common in patients without AWS, but the difference was not significant [p = 0.156]. The average BMI equaled 28.15 ± 0.98 in patients without AWS and 27.29 ± 1.05 for those with AWS and this difference was not significant [Figure 2].

Exploring the hypothesis that the aforementioned variables could contribute to the onset of AWS, a multivariate analysis was conducted. Weak correlations were observed between the variables of age, BMI, time interval between treatment and data collection, surgical team, number of lymph nodes removed and type of surgery and the presence of AWS, as the Kaiser-Meyer-Olkin coefficient equaled 0.489, associated with a p value of less than 0.001, in the Bartlet test of sphericity. From the analysis of the communalities and from the normal and rotated variance matrices, it was found that the risk of triggering the syndrome increased at a younger age (21.7%), greater time interval between the first treatment and data collection (29.3%), greater number of lymph nodes removed (149.7%) and surgical management according to teams (113.2%) [Table 4].

**Table 4:** **Analysis of determinants factors of AWS**

**Variables**	**Load factor**	**Odds-ratio**	**95% CI**	**P Value**
Age in years (younger)	0.596	1.217	1.085-1739	**0.017**
Time between first treatment and data collection (more time)	0.704	1.293	1.011-1.836	**0.046**
Surgical team (less conservative)	0.870	2.132	1.870-5.215	**0.007**
Number of lymph nodes removed (greater number)	0.802	2.497	1.013-6.155	**0.044**

## Discussion

The frequency of axillary web syndrome was 28.86%. This percentage is consistent with other descriptions, in the literature, which vary between 6% and 48.3%, due to imprecision in the information of the definition criterion of the syndrome and the differences in methodology (Moskovitz et al. 2001; Bergmann et al. 2012; Lacomba et al. 2009; Bergmann et al. 2007; Leidenius et al. 2003).

In relation to the criterion of syndrome diagnosis, palpation or visualization of the cords was used by the studies, as well as association with the presence of pain on palpation or reducing the range of motion of the limb ipsilateral to the affected breast. However, these criteria were usually not described in detail, making a more accurate comparison of frequencies impossible (Moskovitz et al. 2001; Bergmann et al. 2012; Lacomba et al. 2009; Bergmann et al. 2007; Leidenius et al. 2003).

The difference in the type of study may have contributed to the variability in percentages of occurrence of AWS. Exclusively retrospective research identified a lower frequency of the syndrome (Moskovitz et al. 2001), whereas prospective studies, with the start of the preoperative and follow-up period varying between 45 days and two years, reporting higher frequencies (Bergmann et al. 2012; Lacomba et al. 2009; Bergmann et al. 2007; Leidenius et al. 2003).

The frequency of AWS in patients of this research should be analyzed with caution since most of them had not started chemotherapy or radiotherapy at the time of data collection and, additionally, for those who made them, the time of internation was short. It follows that the possibility of developing AWS later (Lacomba et al. 2009; Leidenius et al. 2003) could not be excluded, although reports from the literature agree that the cords usually make their appearance in the first three months after surgery (Koehler 2009; Lacomba et al. 2009). Therefore, it was important in this study to indentify precocious appearance of AWS, more frequent within the first 60 days, as described by several studies (Lacomba et al. 2009; Leduc et al. 2009; Fourie & Robb 2009; Whitley & Ball 2002; Bergmann et al. 2007; Leidenius et al. 2003).

Not relationship was observed between the presence of AWS and lateralization of the breast affected by breast cancer, as also reported by Bergmann et al. (2012). Additionally, in the case of clinical stage, there was no association between the advanced stages and the presence of AWS, corroborating some recent studies (Bergmann et al. 2012; Bergmann et al. 2007). The apparent contradiction of this finding could derive from the transversality of the present study having served as a possible factor of underestimation in the detection of AWS, as the installation of AWS could occur at another time during the treatment or even after treatment.

Despite the initial stage having prevailed, there was a greater number of patients who underwent modified radical mastectomy, reflecting the surgical option of hospital service teams where the survey was conducted. This hypothesis seems to be corroborated by the fact that women without the syndrome were also predominantly given this treatment.

It is relevant to note that the surgical team acted as an increased risk factor for triggering AWS in the multivariate analysis, indicating that, in addition to the preference for modified radical mastectomy, there was also a greater tendency for axillary dissection, even considering the predominance of patients being in the initial clinical stage. This association has been reported in other Brazilian studies (Bergmann et al. 2012; Bergmann et al. 2007), unlike foreign research, in which conservative surgeries predominated for initial stages (Lacomba et al. 2009; Leidenius et al. 2003).

The average number of axillary lymph nodes removed found in this research agrees with the findings of Bergmann et al. (Bergmann et al. 2012), who reported a predominance of 15 or more lymph nodes removed. It is worth emphasizing that the number of lymph nodes removed acted as a contributing factor for the increase in the risk of developing AWS when multivariate analysis was performed in this study, therefore agreeing with other studies (Bergmann et al. 2012; Bergmann et al. 2007).

Still in relation to axillary lymph nodes, the results of the present research agreed with Bergmann et al. (2007) in indicating a higher number of lymph nodes involved (metastatic) in patients without AWS, when compared to those with AWS. The importance of the number of positive lymph nodes seen in histopathological examination lies in the increase of 13% to 62% in the risk of developing AWS (Bergmann et al. 2012; Lacomba et al. 2009; Bergmann et al. 2007; Leidenius et al. 2003). However, in the present research, this finding was not confirmed, which is in agreement with the study by Bergmann et al. (Bergmann et al. 2007).

An interesting observation is that one-third of patients submitted to examination for sentinel lymph node, without axillary dissection, developed AWS. This leads us to reflect that even small interventions in the armpit could cause major and clinically apparent injuries, which is in accordance with the review published by Fukushima, Silva and Ferreira (2011).

The present study did not detect a relationship between BMI and AWS, contrary to the research of (Bergmann et al. 2012), in which obese women showed a reduction of 15% in the risk for AWS (although without statistical significance). These authors published, in 2007, research that showed a higher frequency of women with AWS in thin patients (Bergmann et al. 2007). The sample composition of the present study, with the largest number of women above BMI 25, may have favored a greater frequency of AWS in overweight or obese women.

The main limitation of this research was the transversality, i.e. no follow-up of patients over time and thus conclusions of causality of predisposing factors to axillary web syndrome could not be determined. Furthermore, the retrospective step constitutes, per se, a limitation, since information written by third parties and by self-reporting of symptoms experienced in the past by the same patients may lead to bias of interpretation and memory.

The present research did not evaluate the patients in the period prior to surgery, as in some studies (Bergmann et al. 2007; Gärtner et al. 2009; Petrek et al. 2001; Sagen et al. 2009), which, in turn, did not confirm the likely post-surgical etiology of AWS.

## Conclusions

Axillary web syndrome was diagnosed in 28.86% of the women. The risk of triggering the syndrome were increased for younger age (21.7%), longer time between the first treatment and data collection (29.3%), a greater number of lymph nodes removed (149.7%) and surgical management according to medical teams (113.2%).

Further studies are needed to evaluate prior-to-surgery and longer post-operative follow-up, to detect predisposing factors and adequately characterize AWS. Additionally, greater importance should be given to the effects of involving the axilla in surgery on homeostatic balance, not just in the ipsilateral upper limb, but also in the whole organism, so that preventive and therapeutic interventions can be optimized.

## Abbreviations

CHP: Cancer Hospital of Pernambuco
BMI: Body mass index
SMRS: Service of medical records and statistics
AWS: Axillary web syndrome

## References

[CR1] Bergmann A, Mattos IE, Pedrosa E, Nogueira EA, Koifman RJ (2007). Axillary web syndrome after lymph node dissection: results of 1004 breast cancer patients. Lymphology.

[CR2] Bergmann A, Mendes VV, Dias RA, Silva BAS, Ferreira MGCL, Fabro EAN (2012). Incidence and risk factors for axillary web syndrome after breast cancer surgery. Breast Cancer Res Treat.

[CR3] Biazús JV, Artes Médicas S (2000). Mama e Técnica do Linfonodo-Sentinela. Rotinas em Cirurgia Conservadora da Mama.

[CR4] Brasil. Instituto Nacional do Câncer Jose Alencar Gomes da Silva. Coordenação Geral de Ações Estratégicas (2011) Coordenação de Prevenção e Vigilância: Estimativa 2012: incidência de câncer no Brasil.

[CR5] Canadian Cancer Society (2012). Canadian Cancer Society’s Steering Committee On Cancer Statistics 2012.

[CR6] De Martino RR, Goodney PP, Spangler EL, Wallaert JB, Corriere MA, Rzucidio EM, Walsh DB, Stone DH (2012). Variation in thromboembolic complications among patients undergoing commonly performed cancer operations. J Vasc Surg.

[CR7] Ewertz M, Jensen AB (2011). Late effects of breast cancer treatment and potentials for rehabilitation. Acta Oncol.

[CR8] Fourie WJ, Robb KA (2009). Physiotherapy Management of Axillary Web Syndrome Following Breast Cancer Treatment: Discussing the Use of Soft Tissue Techniques. Physiotherapy.

[CR9] Freitas-Júnior R, Gonzaga CMR, Freitas NMA, Matins E, Dardes RCM (2012). Disparities in female breast cancer mortality rates in Brazil between 1980 and 2009. Clinics.

[CR10] Fukushima KFP, Silva HJ, Ferreira CWS (2011). Alterações vasculares resultantes da abordagem cirúrgica da axila: Uma revisão da literatura. Rev Bras de Mastologia.

[CR11] Gärtner R, Jensen MB, Nielsen J, Ewertz M, Kroman N, Kehlet H (2009). Prevalence of and factors associated with persistent pain following breast cancer surgery. JAMA.

[CR12] Hellman S, Harris JR (2002). História Natural Do Câncer De Mama. Doenças da Mama.

[CR13] Isaksson G, Feuk B (2000). Morbidity from axillary treatment in breast cancer: a follow-up study in a district hospital. Acta Oncol.

[CR14] Koehler LA, Zuther JE (2009). Axillary Web Syndrome. Lymphedema Management: The Comprehensive Guide for Patients and Practitioners.

[CR15] Lacomba MT, Mayoral Del Moral O, Coperias Zazo JL, Yuste Sánchez MJ, Ferrandez JC, Zapico GA (2009). Axillary web syndrome after axillary dissection in breast cancer: a prospective study. Breast Cancer Res Treat.

[CR16] Leduc O, Sichere M, Moreau A, Rigolet J, Darc S, Wilputte F, Strapart J, Parijs T, Clément A, Snoeck T, Pastouret F, Leduc A (2009). Axillary web syndrome: nature and localization. Lymphology.

[CR17] Leidenius M, Leppänen E, Krogerus L, Von Smitten K (2003). Motion restriction and axillary web syndrome after sentinel node biopsy and axillary clearance in breast cancer. Am J Surg.

[CR18] Lovely JK, Nehring RN, Boughey JC, Degnim AC, Donthi R, Harmsen WS, Jakub JW (2012). Balancing Venous thromboembolism and hematoma after breast surgery. Ann Surg Oncol.

[CR19] Moskovitz AH, Anderson BO, Yeung RS, Byrd DR, Lawton TJ, Moe RE (2001). Axillary web syndrome after axillary dissection. Am J Surg.

[CR20] Petrek JA, Senie RT, Peters M, Rosen PP (2001). Lymphedema in a cohort of breast carcinoma survivors 20 years after diagnosis. Cancer.

[CR21] Sagen A, Karesen R, Sandvik L, Risberg MA (2009). Changes in arm morbidities and health- related quality of life after breast cancer surgery – a five-year follow-up study. Acta Oncol.

[CR22] Siegel R, Naishadham D, Jemal A (2012). Cancer statistics 2012 American Cancer Society. CA Cancer J Clin.

[CR23] Springer BA, Levy E, Garvey C, Pfalzer LA, Stout NL, Gerber LH, Soballe PW, Danoff J (2010). Pre-operative assessment enables early diagnosis and recovery of shoulder function in patients with breast cancer. Breast Cancer Res Treat.

[CR24] Steegers MA, Wolters B, Evers AW, Strobbe L, Wilder-Smith OH (2008). Effect of axillary lymph node dissection on prevalence and intensity of chronic and phantom pain after breast cancer surgery. J Pain.

[CR25] Tasmuth T, von Smitten K, Hietanen P, Kataja M, Kalso E (1995). Pain and other symptoms after different treatment modalities of breast cancer. Ann Oncol.

[CR26] Whitley E, Ball J (2002). Statistics review 4: sample size calculations. Crit Care (Bethesda).

